# Estimating the effects of lockdown timing on COVID-19 cases and deaths in England: A counterfactual modelling study

**DOI:** 10.1371/journal.pone.0263432

**Published:** 2022-04-14

**Authors:** Kellyn F. Arnold, Mark S. Gilthorpe, Nisreen A. Alwan, Alison J. Heppenstall, Georgia D. Tomova, Martin McKee, Peter W. G. Tennant

**Affiliations:** 1 Leeds Institute for Data Analytics, University of Leeds, Leeds, United Kingdom; 2 Faculty of Environment, University of Leeds, Leeds, United Kingdom; 3 Faculty of Medicine and Health, University of Leeds, Leeds, United Kingdom; 4 The Alan Turing Institute, London, United Kingdom; 5 School of Primary Care, Population Sciences and Medical Education, Faculty of Medicine, University of Southampton, Southampton, United Kingdom; 6 NIHR Southampton Biomedical Research Centre, University of Southampton and University Hospital Southampton NHS Foundation Trust, Southampton, United Kingdom; 7 NIHR Applied Research Collaboration (ARC) Wessex, Southampton, United Kingdom; 8 School of Social and Political Sciences, MRC/CSO Social and Public Health Sciences Unit, University of Glasgow, Glasgow, United Kingdom; 9 London School of Hygiene and Tropical Medicine, London, United Kingdom; Centers for Disease Control and Prevention, UNITED STATES

## Abstract

**Background:**

During the first wave of the COVID-19 pandemic, the United Kingdom experienced one of the highest per-capita death tolls worldwide. It is debated whether this may partly be explained by the relatively late initiation of voluntary social distancing and mandatory lockdown measures. In this study, we used simulations to estimate the number of cases and deaths that would have occurred in England by 1 June 2020 if these interventions had been implemented one or two weeks earlier, and the impact on the required duration of lockdown.

**Methods:**

Using official reported data on the number of Pillar 1 lab-confirmed cases of COVID-19 and associated deaths occurring in England from 3 March to 1 June, we modelled: the *natural* (i.e. observed) growth of cases, and the *counterfactual* (i.e. hypothetical) growth of cases that would have occurred had measures been implemented one or two weeks earlier. Under each counterfactual condition, we estimated the expected number of deaths and the time required to reach the incidence observed under natural growth on 1 June.

**Results:**

Introducing measures one week earlier would have reduced by 74% the number of confirmed COVID-19 cases in England by 1 June, resulting in approximately 21,000 fewer hospital deaths and 34,000 fewer total deaths; the required time spent in full lockdown could also have been halved, from 69 to 35 days. Acting two weeks earlier would have reduced cases by 93%, resulting in between 26,000 and 43,000 fewer deaths.

**Conclusions:**

Our modelling supports the claim that the relatively late introduction of social distancing and lockdown measures likely increased the scale, severity, and duration of the first wave of COVID-19 in England. Our results highlight the importance of acting swiftly to minimise the spread of an infectious disease when case numbers are increasing exponentially.

## Introduction

On 11 March 2020, the World Health Organisation (WHO) declared COVID-19 a global pandemic [1]. In the three months since it emerged, the virus had infected over 100,000 people worldwide. While much remained unknown, it was clear the virus had a high potential for transmission and induced substantial morbidity and mortality. Without proactive intervention, the number of COVID-19 cases appeared to increase exponentially [2, 3], consistent with a novel infection with a basic reproduction number (i.e. R_0_) above 1 [4].

In the absence of a vaccine or suitable pharmacological interventions, reducing the spread of COVID-19 relies on public health interventions such as contact tracing and ‘social distancing’. Unfortunately, few countries had sufficient initial capacity to control their outbreaks through testing, tracing, and isolation alone. Most governments therefore relied on population-wide measures, often in the form of a national ‘lockdown’ [5, 6].

In Europe, the first lockdown was imposed in Italy on 9 March 2020, followed by Ireland (12 March), Albania and Poland (13), Spain (14), Serbia (15), and Austria, the Czech Republic, and Lithuania (16). In England, several voluntary social distancing measures (e.g. limits on mass gatherings and social contact) were introduced on 17 March, but a mandatory lockdown (including closure of schools and non-essential shops, public transport restrictions, and limits on time outside the home) was not enforced until 24 March [7, 8]. By 1 June, just ten weeks later, approximately 220,000 people living in England had tested positive for the virus [9], with 45,000 COVID-19 deaths reported [10]. The United Kingdom (UK) as a whole has suffered one of the highest per capita death tolls worldwide [11].

The extent that England’s later lockdown might have contributed to high COVID-19 mortality is contested. In a pair of interviews on 7 June, Prof John Edmunds of the UK’s Scientific Advisory Group for Emergencies (SAGE) declared the delay likely ‘cost a lot of lives’ [12]; this was disputed by Health Secretary Matt Hancock, who asserted the government ‘took the right decisions at the right time’ [13]. Nevertheless on 10 June, another (former) member of SAGE, Prof Neil Ferguson, claimed that deaths could have been halved if lockdown had been enacted a week earlier [14].

Some attempts have been made to quantify the effects of lockdown timing on cases and deaths, both within the UK and elsewhere [15–22]. Oftentimes, such studies utilise compartmental SEIR (i.e. Susceptible, Exposed, Infected, Recovered) models, an equation-based approach in which a simulated population is subdivided into compartments based on health status, the sizes of which evolve over time according to specified parameters [23]. In the UK, for instance, Dagpunar [15] estimated that intervening one week earlier would have reduced deaths during the first wave by 72%, preventing approximately 28,000 deaths; Dropkin [16] estimated a similar figure, with 30,000 fewer deaths by 9 June. In the United States, Pei et al. [18] estimated that 57% of reported infections and 54% of reported deaths could have been averted by 3 May if measures had been implemented one week earlier, with both figures increasing to 89% for two weeks earlier. Worldwide, Papadopoulos et al. [24] found that earlier social distancing measures were associated with reduced COVID-19 mortality.

Quantifying the impact of England’s later lockdown is important for improving our understanding of the effects of lockdown-style measures, including how and when they offer maximum benefit. However, estimates from SEIR models (and more complex agent-based models [23]) often attract scepticism due to their reliance on, and sensitivity to, assumptions about unknown features of the infection and disease processes (e.g. duration of infectivity, basic reproduction number R_0_) [25]. We therefore use a simpler approach that directly models the causal process of exponential growth, to estimate the reduction in confirmed COVID-19 cases and deaths that would have occurred in England by 1 June had public health interventions been introduced one or two weeks earlier. We also estimate the required time spent in lockdown to reach the same number of incident cases as observed on 1 June.

## Methods

Exponential growth represents an inherently causal process, whereby the number of new cases on a given day (*t*) is a multiple (*r*) of the number of existing cases. This is analogous to the relationship between the number of infectious cases, reproduction number, and number of new cases, but the growth factor *r* carries less direct meaning. Nevertheless, the effects of systemic interventions should be similarly reflected in changes in the growth factor as by changes in the reproduction number. This permits estimation of how the growth of cases *would have been different* under different conditions, without needing to impose uncertain yet common assumptions.

We considered the growth of COVID-19 cases in England between 3 March and 1 June (i.e. 1≤*t*≤*T*). Data sparsity at the beginning of the epidemic has the potential to adversely affect estimation of growth parameters, since random fluctuations may appear magnified when numbers are small. Therefore, we chose 3 March as it represents the first full day for which cumulative lab-confirmed cases exceeded 100. We chose 1 June as the end of ‘full’ lockdown, since several important restrictions were eased on this day (e.g. reopening of primary schools and permissible household mixing) [26, 27]. For our primary analysis, we modelled both the *natural* growth of confirmed COVID-19 cases over this time period and the *counterfactual* growth resulting from the observed sequence of recommended social distancing and mandatory lockdown measures (on 17 and 24 March, respectively) being implemented one or two weeks earlier. As secondary analyses, we estimated the cumulative number of deaths expected on 1 June and the required time spent in full lockdown to reach the mean number of confirmed incident cases on 1 June under each condition. Analyses were conducted in R (v. 4.0.0) [28]; code is provided in (S1 Appendix).

### Data

Time series data pertaining to incident and cumulative lab-confirmed cases of COVID-19 in England were obtained via the UK government’s website [9], which include cases identified through four testing ‘pillars’ (additional details and description in S1 Appendix). For our analyses, we considered only Pillar 1 data, which consist of testing done by Public Health England (PHE) labs and National Health Service (NHS) hospitals for those with a clinical need and health/care workers; these data provide the most stable (if incomplete) indication of the underlying rate of infection since the criteria and capacity for Pillar 1 testing remained relatively stable across the study period [29]. Cases are attributed to the date of testing.

Time series data pertaining to confirmed COVID-19-associated deaths in England were obtained from two sources:

1. **NHS England**, which reports deaths occurring in *hospitals* for which the patient either tested positive for COVID-19 or where COVID-19 was mentioned on the death certificate [30].
2. **Office for National Statistics (ONS)**, which reports *all* registered deaths for which COVID-19 was mentioned on the death certificate [10].

Deaths are attributed to the date of death.

All data were accessed on 22 July 2020 and are therefore likely to be largely complete for the period examined. Additional data notes are provided in (S1 Appendix) and referenced materials.

### Identification of growth periods and parameters

The equation *X*_*t*_ = *X*_*t*−1_∙*r* is satisfied for a variable *X* which depends exponentially on time *t*. Assuming COVID-19 exhibits exponential growth in the early stages of the pandemic, we may therefore write ${Cumulative\;cases}_{t}={Cumulative\;cases}_{t-1}∙r$, which may be rearranged as:

$${Incident\;cases}_{t}={Cumulative\;cases}_{t-1}∙(r-1)$$ (Eq 1)

Eq 1 implies a linear relationship between cumulative and incident cases over time that should approximate an unperturbed straight line in the absence of exogenous interventions. Social distancing and lockdown measures are likely to cause discernible changes in the slope of this line, resulting in three (relatively) distinct periods of growth:

1. Initial uncontrolled growth;
2. Growth under social distancing;
3. Growth under lockdown.

To identify these periods, we considered the following spline model:

$$\begin{array}{ll} {Incident\;cases}_{t}=\alpha_{1}{Cumulative\;cases}_{t-1}, & 1<t\leq a;\\ {Incident\;cases}_{t}=\beta_{0}+\beta_{1}{Cumulative\;cases}_{t-1}, & a<t\leq b;\\ {Incident\;cases}_{t}=\gamma_{0}+\gamma_{1}{Cumulative\;cases}_{t-1}, & b<t\leq T; \end{array}$$ (Eq 2)

where knot dates *a* and *b*, respectively, represent the points at which the effects on the growth factor *r* of social distancing and lockdown measures were *realised*. The coefficients *α*_1_, *β*_1_, and *γ*_1_ are equal to *r*−1 (from Eq 1), and thus may be used to directly estimate the growth factor.

Delays in symptom onset and testing mean that *a* and *b* are unlikely to coincide with implementation of their respective measures. We therefore allowed for lags of up to 21 days for the effect of each measure on the growth rate to become visible. We considered all possible pairs of knot dates (*a*, *b*) for which *a*<*b*.

For each pair of candidate knot dates (*a*, *b*), we fit the spline model in Eq 2. We accounted for dependencies between observations by including a first-order autoregressive term and accommodated apparent ‘weekend effects’ by specifying a seven-day seasonal period [31]. We then estimated how well the given knot dates and associated growth factors predicted the observed growth of cases between 3 March and 1 June. For each day *t*, 1≤*t*≤*T*, the estimated growth factor $\hat{r}$ was applied to the number of incident cases on the previous day according to the growth period in which it fell, to estimate the number of incident cases on the current day. The Poisson deviance between the observed (7-day moving average) and predicted incident and cumulative cases over the entire period was calculated [32].

Pairs of knot dates which minimised the Poisson deviance with respect to both incident and cumulative cases were retained, and a likelihood-based probability of each pair was constructed.

Additional methodological details are provided in (S1 Appendix).

### Modelling natural and counterfactual growth

We used a stochastic simulation to estimate the growth of COVID-19 cases from 3 March (*t* = 1) to 1 June (*t* = *T*) under three scenarios:

1. Natural growth: social distancing and lockdown as implemented;
2. Counterfactual growth: social distancing and lockdown one week earlier;
3. Counterfactual growth: social distancing and lockdown two weeks earlier.

Each scenario was simulated 100,000 times; the mean number of cumulative cases on 1 June across all simulation runs was calculated with 95 percent simulation interval (SI, i.e. 2.5 and 97.5 centile estimates). To account for uncertainty in the true knot dates, all pairs identified as most likely were used, with their frequencies corresponding to their relative likelihood.

We first describe the natural growth model and then modifications for counterfactual growth models.

For each day *t*, 1≤*t*≤*T*, a random growth factor was drawn from a log-normal distribution according to the growth period in which it fell. Means and standard deviations were estimated from the parameters of the spline equation corresponding to the given knot dates (i.e. coefficients ${\hat{\alpha }}_{1},{\hat{\beta }}_{1}$, and ${\hat{\gamma }}_{1}$ and their standard errors (SEs)); values were estimated on the normal scale and then transformed. The random growth factor was multiplied by the number of incident cases on the previous day (i.e. *t*−1) to calculate the number of incident cases on the current day. At time *T*, the total number of cumulative cases was recorded.

The counterfactual growth models proceeded similarly, but each knot date pair (*a*, *b*) was replaced with (a′,b′)=(a−7,b−7) and (a′,b′)=(a−14,b−14), respectively. For the natural growth model, the Poisson deviance between the observed (7-day moving average) and predicted incident and cumulative cases over the entire period was calculated.

### Estimated deaths

The case fatality ratio (CFR) for COVID-19 is the proportion of deaths attributable to COVID-19 among those diagnosed over a given period (i.e. ${Cumulative\;deaths}_{t}/{Cumulative\;cases}_{t}$). Since the reported number of deaths in England differs considerably between different sources, we calculated two separate CFRs on 1 June (*t* = *T*):

- *CFR*_1_: considers COVID-19-related deaths occurring *in hospitals* (i.e. utilising data from NHS England [30]); and
- *CFR*_2_: considers *all* COVID-19-related deaths (i.e. utilising data from ONS [10]).

At the end of each simulation run, each estimated CFR was applied to the cumulative number of cases to estimate the number of deaths on 1 June; mean and 95 percent SIs across all runs were calculated.

### Lockdown duration

We estimated the mean number of incident cases on 1 June under the natural history. For each counterfactual history, we then calculated the number of days from the initiation of lockdown for the mean number of incident cases to fall below this threshold.

### Sensitivity analyses

We explored the sensitivity of our results to the exclusion of cases identified by the government’s Pillar 2 (i.e. community) testing by repeating all analyses using cases identified through both Pillars 1 *and* 2. We also explored sensitivity to the construction of our likelihood-based probability of each knot date pair by conducting simulations with equal probabilities.

## Results

### Identification of growth periods and parameters

**Fig 1:**
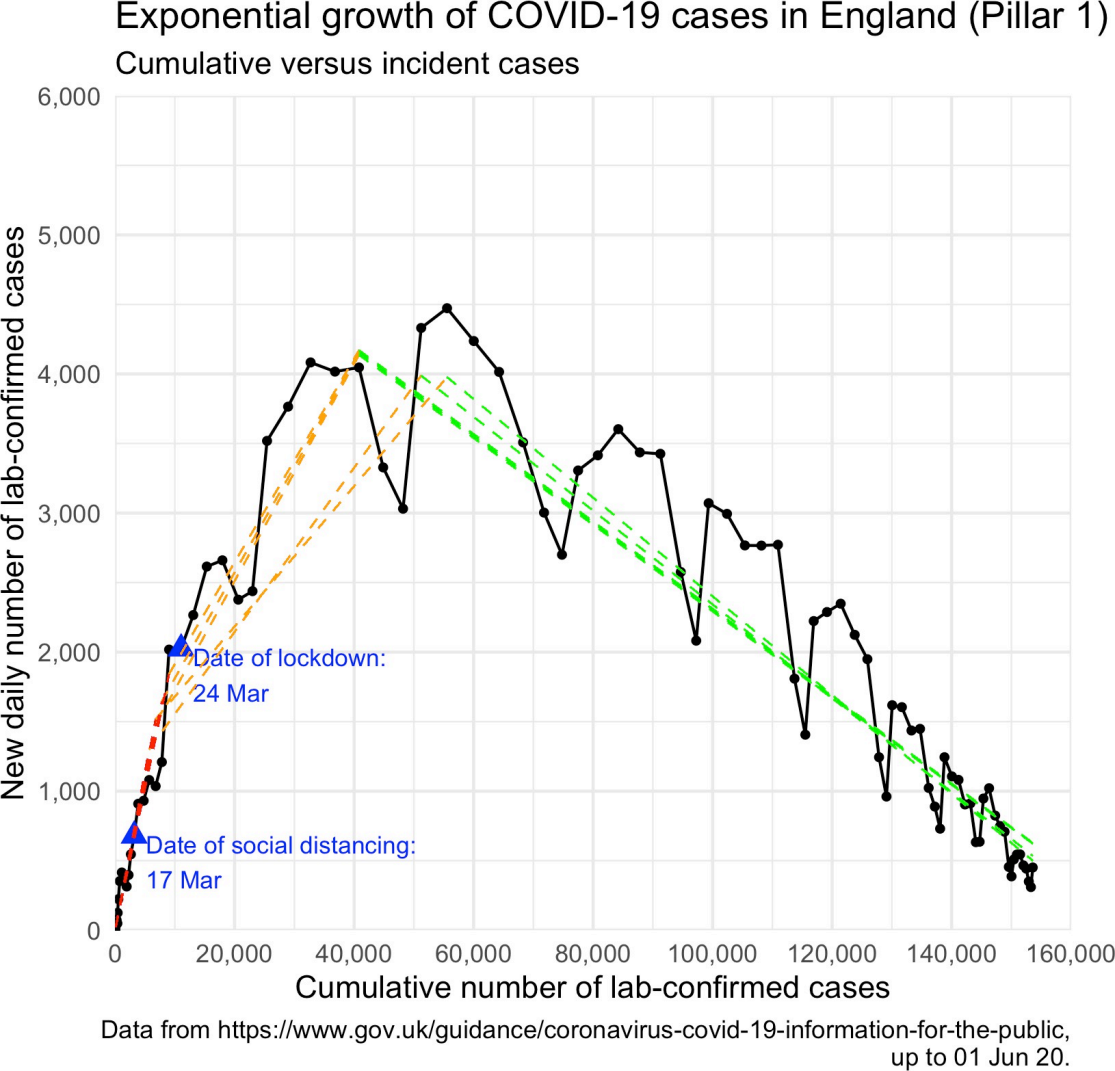
Observed relationship between cumulative and daily number of COVID-19 cases in England. The observed relationship between cumulative and daily number of new Pillar 1 lab-confirmed cases in England from 30 January to 1 June, with the five best-fitting spline models overlaid (see Table 1). Three distinct growth periods are identified, which may reasonably be attributed to measures implemented by the UK government. Growth period 1 (in red) indicates initial uncontrolled growth; growth period 2 (in orange) indicates growth under social distancing; growth period 3 (in green) indicates growth under lockdown. Dates in blue indicate first full days under social distancing and lockdown measures, respectively.

Fig 1 displays the observed relationship between cumulative and daily number of new Pillar 1 cases in England from 30 January to 1 June. The relationship follows a positive and approximately linear trend until cumulative cases reach approximately 40,000 –around which point the trend reverses.

Table 1 summarises the knot date pairs deemed most likely by our analyses, including the estimated growth factors (and SEs) for each growth period and the likelihood-based probability of each pair. Our analyses indicate that initial uncontrolled growth lasted until 20–23 March, where cases increased by a factor of approximately 1.21 each day. The second growth period (following voluntary social distancing measures) lasted until 3–7 April, during which cases increased by a smaller factor of approximately 1.06. In the third growth period (following mandatory lockdown measures), the growth factor fell to approximately 0.96.

**Table 1:** Most likely knot date pairs.

Knot date 1 (*a*)	Knot date 2 (*b*)	Growth factor 1 (*α*_1_+1) [SE]	Growth factor 2 (*β*_1_+1) [SE]	Growth factor 3 (*γ*_1_+1) [SE]	Poisson deviance, incident cases	Poisson deviance, cumulative cases	Prob.
20 March	6 April	1.228 [0.059]	1.059 [0.008]	0.966 [0.005]	2752	5059	0.148
21 March	3 April	1.218 [0.034]	1.078 [0.008]	0.969 [0.003]	2949	2851	0.263
21 March	7 April	1.224 [0.052]	1.050 [0.008]	0.965 [0.006]	2355	3619	0.207
22 March	3 April	1.210 [0.028]	1.076 [0.007]	0.969 [0.003]	2829	2539	0.295
23 March	3 April	1.204 [0.024]	1.073 [0.007]	0.969 [0.003]	2721	8586	0.097

Best-fitting pairs of knot dates, with corresponding growth factors (standard errors, SEs) estimated from Eq 2; all values are given on the normal scale. Poisson deviance with respect to both incident and cumulative cases for the period 1≤t≤T are also given. The likelihood-based probability (Prob.) of each pair of knot dates is also given (calculation described in greater detail in S1 Appendix).

The spline models corresponding to the knot date pairs in Table 1 are overlaid on Fig 1, offering a visual summary of the distinct growth periods.

### Modelling natural and counterfactual growth

**Fig 2:**
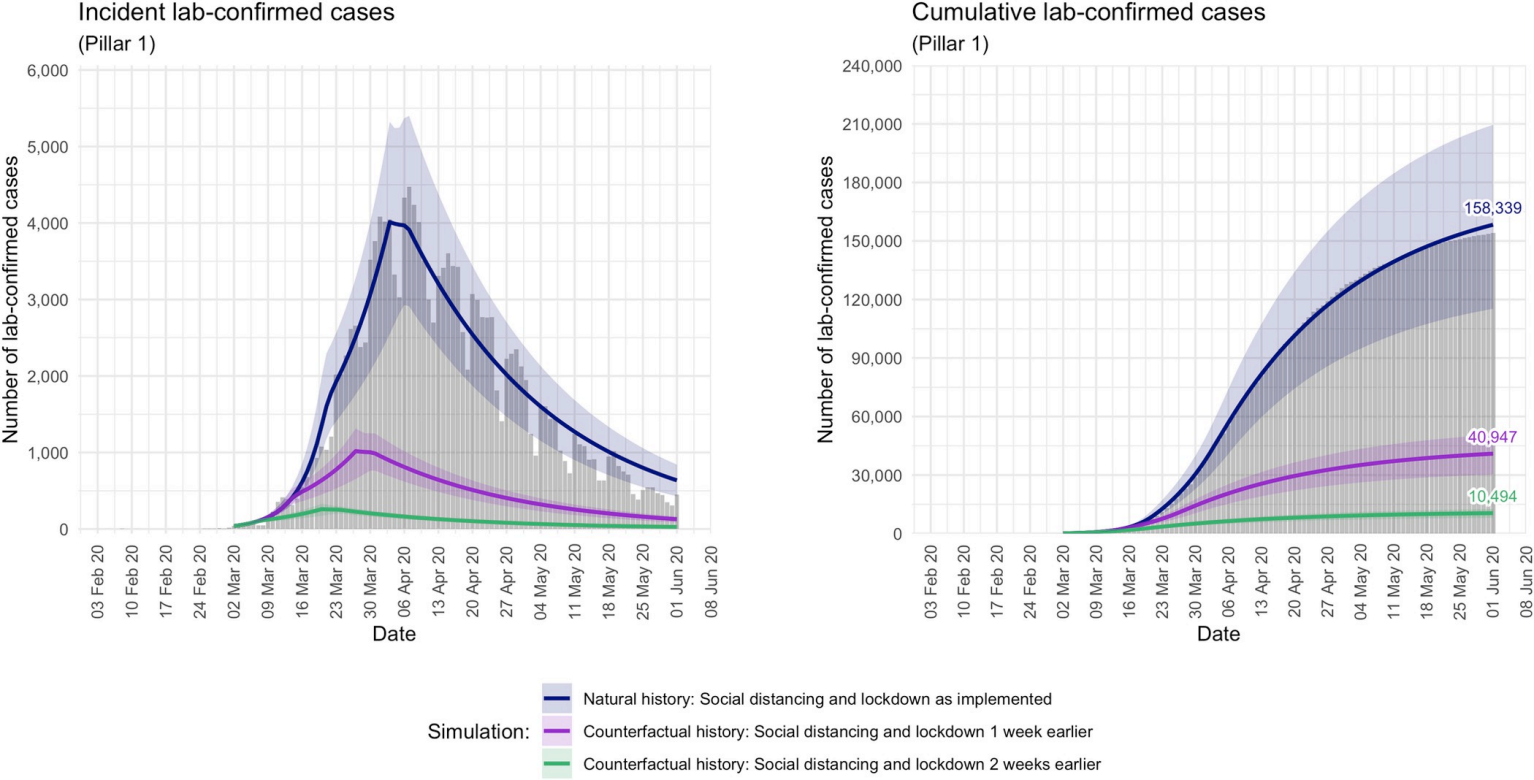
Incident and cumulative COVID-19 cases in England. Incident and cumulative Pillar 1 lab-confirmed cases of COVID-19 from 3 March to 1 June, under each of the three scenarios modelled. Under the natural history, social distancing and lockdown measures were implemented on 17 and 24 March, respectively. Grey bars represent daily number of observed incident cases. Blue lines represent mean number of incident cases across all 100,000 simulation runs, including the 95% simulation interval.

Fig 2 displays incident and cumulative Pillar 1 cases of COVID-19 under the three scenarios of natural growth, counterfactual growth acting one week earlier, and counterfactual growth acting two weeks earlier.

The observed and estimated mean number (95% SI) of cumulative lab-confirmed cases on 1 June for each scenario is given in Table 2. Our simulations suggest that implementing social distancing and lockdown one week earlier would have resulted in a 74% reduction in the total number of Pillar 1 cases, from 158,339 to 40,947, whereas two weeks earlier would have resulted in a 93% reduction, to 10,494.

**Table 2:** Total number of COVID-19 cases for three simulated scenarios.

Cumulative number of lab-confirmed cases (1 June)			
Natural growth		Counterfactual growth	
**Observed**	**Modelled**	**Intervention 1 week earlier**	**Intervention 2 weeks earlier**
154,027	158,339 (115,215, 209,544)	40,947 (30,317, 50,636)	10,494 (8,033, 12,571)

Cumulative number of Pillar 1 lab-confirmed cases of COVID-19 in England on 1 June for each scenario modelled. The mean number of cases from 100,000 simulation runs are given for the three modelled scenarios, with 95% simulation intervals (i.e. 2.5 and 97.5 centile estimates) in parentheses.

As evident in Fig 2, our natural growth model slightly overestimates incident cases in the final growth period, leading to an overestimation of cumulative cases by 1 June of approximately 3%. The Poisson deviance of this model with respect to incident cases is 2,487, and 2,397 with respect to cumulative cases.

### Estimated deaths

CFRs for COVID-19-related deaths occurring either in hospital (i.e. *CFR*_1_) or across all settings (i.e. *CFR*_2_) were estimated to be 0.117 and 0.293, respectively.

The estimated mean number (95% SI) of cumulative deaths on 1 June for each scenario is given in Table 3. Our simulations estimate that 20,740 hospital deaths or 34,385 total deaths could have been avoided had measures been implemented one week earlier; if implemented two weeks earlier, 26,120 hospital deaths and 43,318 total deaths could have been avoided.

**Table 3:** Total number of COVID-19 deaths for three simulated scenarios.

Cumulative number of deaths (1 June)				
	Natural growth		Counterfactual growth	
**Deaths (*CFR*)**	**Observed**	**Modelled**	**Intervention 1 week earlier**	**Intervention 2 weeks earlier**
Hospital deaths (*CFR*_1_)	27,212	27,974 (20,355, 37,020)	7,234 (5,356, 8,946)	1,854 (1,419, 2,221)
All deaths (*CFR*_2_)	45,130	46,393 (33,758, 61,397)	11,998 (8,883, 14,836)	3,075 (2,354, 3,683)

Cumulative number of deaths resulting from COVID-19 in England on 1 June for each scenario modelled. Estimates are given according to two separate case fatality ratios: *CFR*_1_, which utilises data from NHS England on deaths occurring in hospitals [30]; and *CFR*_2_, which utilises data from ONS on all deaths [10]. The mean number of deaths from 100,000 simulation runs are given for the three modelled scenarios, with 95% simulation intervals (i.e. 2.5 and 97.5 centile estimates) in parentheses.

### Lockdown duration

A mean of 636 incident cases were estimated on 1 June (i.e. after 69 days of ‘full’ lockdown). The same threshold would have been reached after just 35 days if measures had been implemented one week earlier, and would never have been exceeded if measures had been implemented two weeks earlier.

### Sensitivity analyses

The implications were unchanged when models included Pillar 2 cases or used equal probabilities for each knot point pair (see S1 Appendix).

## Discussion

We estimated the number of confirmed COVID-19 cases and associated deaths in England that could have been avoided by 1 June if earlier social distancing and lockdown measures had been initiated. Our simulations suggest that nearly three quarters of Pillar 1 cases (largely representing severe cases requiring hospital admission) and approximately 21,000 deaths occurring in hospitals could have been avoided by acting one week earlier; moreover, the time required in lockdown could have been halved (from 69 to 35 days). Although many factors likely contribute to the scale and severity of each COVID-19 epidemic, our modelling suggests that England’s relatively late lockdown may partly explain its comparatively high number of deaths.

### Strengths and limitations

This study is among the first to attempt to formally quantify the impact of the timing of social distancing and lockdown on the number of subsequent COVID-19 cases and deaths experienced by England during the pandemic’s first wave. Our results are broadly consistent with those examining the UK as a whole (e.g. Dagpunar (15), Dropkin (16), and Lander (17)). Our analyses did not rely on forecasting or require assumptions about transmission rates or the basic or effective reproduction numbers; we aimed to bypass the need for such assumptions by directly modelling the underlying causal process, such that the parameters of interest could be directly estimated from observed data. Using stochastic simulations, we were able to account for variation and uncertainty in our estimates with respect to growth rates and time lags between the implementation of interventions the realisation of their effects.

Our modelling nevertheless provides an incomplete summary of the first wave, since we only used data pertaining to Pillar 1 lab-confirmed cases of COVID-19, which are far lower than the true number of infections [33]. Since the number of confirmed cases is extremely dependent upon the level of testing [34, 35], our estimates of absolute number of averted COVID-19 cases are likely to be low. However, the proportion of confirmed cases averted should be fairly robust, since the criteria for Pillar 1 testing remained stable across the study period. Regardless, the reliability of our results is dependent upon the data used, so uncertainty remains about how and to what extent they may be extrapolated to the entire population.

Both CFR calculations are likely to be slight underestimates, since they do not account for the delay between symptom onset and recovery/death [36]. We did not adjust our estimates to take into account distributional estimates between symptom onset and death [37, 38], nor did we take into account variation and/or uncertainty since our primary analysis focussed on the modelling of cases. We assumed that case fatality remained constant across both natural and counterfactual growth scenarios; however, it is plausible that a smaller case load may have afforded the NHS more time and resources to treat individual patients, and further reduced the fatality ratio [39]. Conversely, increased case load may have provided increased opportunity for system learning about best treatment regimes, although any such effect would likely have been modest over the period observed. Moreover, although the characteristics of both cases and deaths vary extensively (e.g. by age, sex), the lack of disaggregated Pillar 1 case data prevented us from accounting for these factors. These considerations together mean that the estimated number of deaths averted should be considered qualitatively less certain than the estimated number of cases.

Estimates relating to deaths occurring in hospitals are likely to be more robust than those across all settings, since there were arguably at least two distinct epidemics under way in England at the time–one in the general population and another in care homes. The measures we examined would likely have been less effective in combating the latter, which were driven by a lack of adequate protective equipment and insufficient protocols for the discharge and transfer of patients between hospitals and care homes.

We assumed that changes in the growth factor directly resulted from the government’s social distancing and lockdown policies. We believe this is a reasonable assumption since the rate of spread in the early stages of an epidemic is unlikely to decrease naturally while the susceptible population remains large. Nevertheless, we did make the simplifying assumption that each intervention produced a sharp (i.e. nondifferentiable) change in the growth factor. In reality, the changes were likely to have been more gradual and/or varied over time (e.g. due to variation in symptom onset or changing adherence to advice). However, the use of multiple pairs of likely knot dates within our simulations allowed for more gradual changes when averaged across all simulation runs.

Finally, our models did not consider any potential effects of the government’s modest easing of some restrictions in mid-May [26].

## Conclusions

“Be fast. Have no regrets.” -Dr Michael Ryan, Executive Director, WHO Health Emergencies Programme [40].

Many potential explanations for high COVID-19 mortality in England and the UK have been debated [41]. Our modelling supports the claim that the relatively late introduction of social distancing and lockdown measures likely increased the scale, severity, and duration of the first wave of COVID-19 in England. Our results suggest that nearly three quarters of confirmed COVID-19 cases in England and approximately 21,000 deaths occurring in hospitals could probably have been avoided by 1 June if social distancing and lockdown measures had been implemented one week earlier, and the required duration of lockdown halved.

Although our modelling is based on empirical observations and various assumptions, our results highlight the importance of acting swiftly to minimise the spread of an infectious disease when case numbers are increasing exponentially. Though common in many settings, the behaviour of exponential series may be underappreciated by public and political figures, particularly where the exponent is substantially above 1 [42]. Although a ‘wait and see’ approach may be more familiar–and indeed reliable for many routine problems–it can prove costly during pandemics. While population immunity to a novel infection remains low, even small delays may quickly lead to substantial numbers of cases and deaths, and ultimately prolong the length of time spent under severe restrictions.

## Supporting information

S1 Appendix Technical appendix. (PDF)
