# Supplementary material for: Estimating the effects of lockdown timing on COVID-19 cases and deaths in England: A counterfactual modelling study
Source: PLoS One. 2022 Apr 14;17(4):e0263432. doi: 10.1371/journal.pone.0263432 (PMC9009677; doi:10.1371/journal.pone.0263432)
Supplement: S1 Appendix — (PDF) [file pone.0263432.s001.pdf]

Supplementary materials

Data

Cases

Time series data relating to incident and cumulative cases of COVID-19 in England were obtained via the UK government's official website. These data are available as a CSV file under the heading 'Time series documents' of the referenced webpage.[1] Data are updated daily and contain cases which have been identified through four different testing 'pillars':

1. Pillar 1: Swab (antigen) testing by Public Health England (PHE) labs and National Health Service (NHS) hospitals for those with a clinical need and health and care workers;
2. Pillar 2: Swab (antigen) testing for the wider population, typically carried out by commercial partners;
3. Pillar 3: Serology testing to show if people have antibodies from previous coronavirus infection; and
4. Pillar 4: Blood and swab testing for national surveillance supported by PHE, the Office for National Statistics (ONS), and research, academic, and scientific partners to learn more about the prevalence and spread of the virus for other testing purposes (e.g. accuracy and ease of use for home testing).

For our analyses, we used only Pillar 1 data, since these provided the most stable indication for the underlying rate of infection.[2] Cases are attributed to the date of testing, rather than the date of confirmation of a positive result. Data are continually updated, meaning that substantial changes are likely to occur for counts reported during the previous 5 days and more minor changes may occur further in the past.

The data used for our analysis were downloaded on 22 July 2020, and which contained data up to 21 July 2020 [source filename: *2020-07-22_COVID-19_UK_positive_cases_time_series_by_specimen_date.csv*]. Therefore, these data are likely to be mostly if not wholly complete for the time period covered by our analyses (i.e. 3 March to 1 June 2020).

Additional data notes are available in referenced material.[1,3]

Deaths

Time series data relating to daily and cumulative COVID-19-associated deaths in England were obtained from two sources:

1. **NHS England**, which reports figures on deaths occurring in *hospitals* for which the patient either tested positive for COVID-19 or where COVID-19 was mentioned on the death certificate.[4] These data are available as an Excel file under the heading 'Data' and subheading 'COVID-19 daily announced deaths' of the referenced webpage.[4] Updated datasets are published every day, meaning that substantial changes are likely to occur for counts reported during the previous 5 days and more minor changes may occur further in the past.

2. **Office for National Statistics (ONS)**, which reports figures on all registered deaths for which COVID-19 was mentioned on the death certificate.[5] These data are available as an Excel file on the referenced webpage.[5] Updated datasets are published weekly.

For both sources, deaths are attributed to the date of death, rather than the date of reporting/announcement.

NHS England data were downloaded on 22 July 2020, which contained data up to 20 July 2020 [source filename: *COVID-19-total-announced-deaths-21-July-2020.xlsx*]. Daily death figures were taken from the tab entitled 'Tab1 Deaths By Region', which contains incident deaths for the whole of England and disaggregated by region. Cumulative death figures were calculated by the authors while preparing the data for subsequent analysis.

ONS data were downloaded on 22 July 2020, which contained data up to and including the 28th week of 2020, i.e. up to 10 July 2020 [source filename: *publishedweek282020.xlsx*]. Cumulative death figures were taken from the tab entitled 'Covid-19 – England comparisons', column 'ONS deaths by actual data of death – registered by 18 July'. Daily death figures were calculated by the authors while preparing the data for subsequent analysis.

Identification of growth periods and parameters

We considered the period between 3 March (i.e. $t = 1$) and 1 June (i.e. $t = T$).

We allowed for a lag of up to 21 days from when social distancing measures began (i.e. 17 March) and when their effects on the growth rate became visible (i.e. knot date a). Similarly, we allowed for a lag of up to 21 days from when lockdown measures began (i.e. 23 March) and when their effects on the growth rate became visible (i.e. knot date b). We considered all possible pairs of knot dates (a, b) for which $a < b$.

For each pair of candidate knot dates (a, b), we fit the following spline model:

$$Incident\ cases_t = \alpha_1 Cumulative\ cases_{t-1}, \qquad 1 < t \leq a$$
$$Incident\ cases_t = \beta_0 + \beta_1 Cumulative\ cases_{t-1}, \qquad a < t \leq b$$
$$Incident\ cases_t = \gamma_0 + \gamma_1 Cumulative\ cases_{t-1}, \qquad b < t \leq T$$

We accounted for dependencies between observations and accommodated apparent 'weekend effects' in data collection (i.e. where fewer COVID-19 tests are administered on the weekends) by fitting an Arima model with one autoregressive term (i.e. $p = 1$) and seven-day seasonal adjustment.[6]

For each pair of candidate knot dates, we recorded the three growth factor corresponding to each of the three spline segments as the slope of the segment (i.e. α_1, β_1, or γ_1) plus one, since $\frac{Incident\ cases_t}{Incident\ cases_{t-1}} = \frac{Cumulative\ cases_{t-1} \cdot (r-1)}{Cumulative\ cases_{t-2} \cdot (r-1)} = \frac{Cumulative\ cases_{t-2} \cdot r}{Cumulative\ cases_{t-2}} = r$. We also recorded the standard deviation (SD) of each growth factor as the estimated standard error for each of α_1, β_1, or γ_1, respectively.

Note that we did not evaluate the spline model directly (e.g. via AIC/BIC), since these measures were determined to be poor calibration statistics. Because of the way that cases increase multiplicatively (i.e. by a factor of r), small overestimations in the growth rate in the first period

correspond to large errors in model predictions (a manifestation of the 'butterfly effect'), which AIC/BIC cannot account for.

Instead, we estimated how well the given knot dates and associated growth factors predicted the observed growth of cases between 3 March and 1 June. For each day t, $1 \leq t \leq T$, the estimated growth factor was applied the number of incident cases on the previous day according to the period of growth in which it fell, in order to calculate the number of incident cases on the current day. The Poisson deviance between the observed (7-day moving average) and predicted incident and cumulative cases over the entire period was calculated;[7] this criteria was selected due to it being a likelihood-based measure that can account for the fact that the model error likely scales with the number of cases.[7]

The 10 'best' pairs of knot points according to each of the following two criteria were calculated: (1) those which produced the lowest Poisson deviance with respect to incident cases, and (2) those which produced the lowest Poisson deviance with respect to cumulative cases. Pairs of knot points which satisfied both criteria were retained. For each of these pairs, we constructed a likelihood-based probability of that pair based on its Poisson deviance with respect to cumulative cases, since all knot point pairs do not fit the data equally well. Because lower deviance values reflect higher likelihood, we took the inverse for each knot point pair and rescaled them so that all values summed to 1.

Calculation of case fatality ratios (CFRs)

The case fatality ratio (CFR) for COVID-19 is defined as the proportion of deaths attributable to COVID-19 among those diagnosed with COVID-19 over a given time period. For example, the CFR on day t can be expressed as $Cumulative\ deaths_t / Cumulative\ cases_t$.

We calculated two separate CFRs on June 1 (i.e. $t = T$) according to two different death counts:

- $\boldsymbol{CFR_1 = 0.177}$. This CFR considers COVID-19-related deaths occurring in hospitals (data from NHS England;[4] see pages 1-2).
- $\boldsymbol{CFR_2 = 0.293}$. This CFR considers all COVID-19-related deaths (data from ONS;[5] see page 2).

We have not adjusted our CFR estimates since they are unlikely to deviate a substantial amount from the true estimates. Figure 1 displays each of CFR_1 and CFR_2 over time, in which it is apparent that the estimates have either begun to or have already levelled off by 1 June. Our estimates are therefore conservative but unlikely to be substantially biased.

We did not take into account variation/uncertainty in the CFRs.

Figure 1

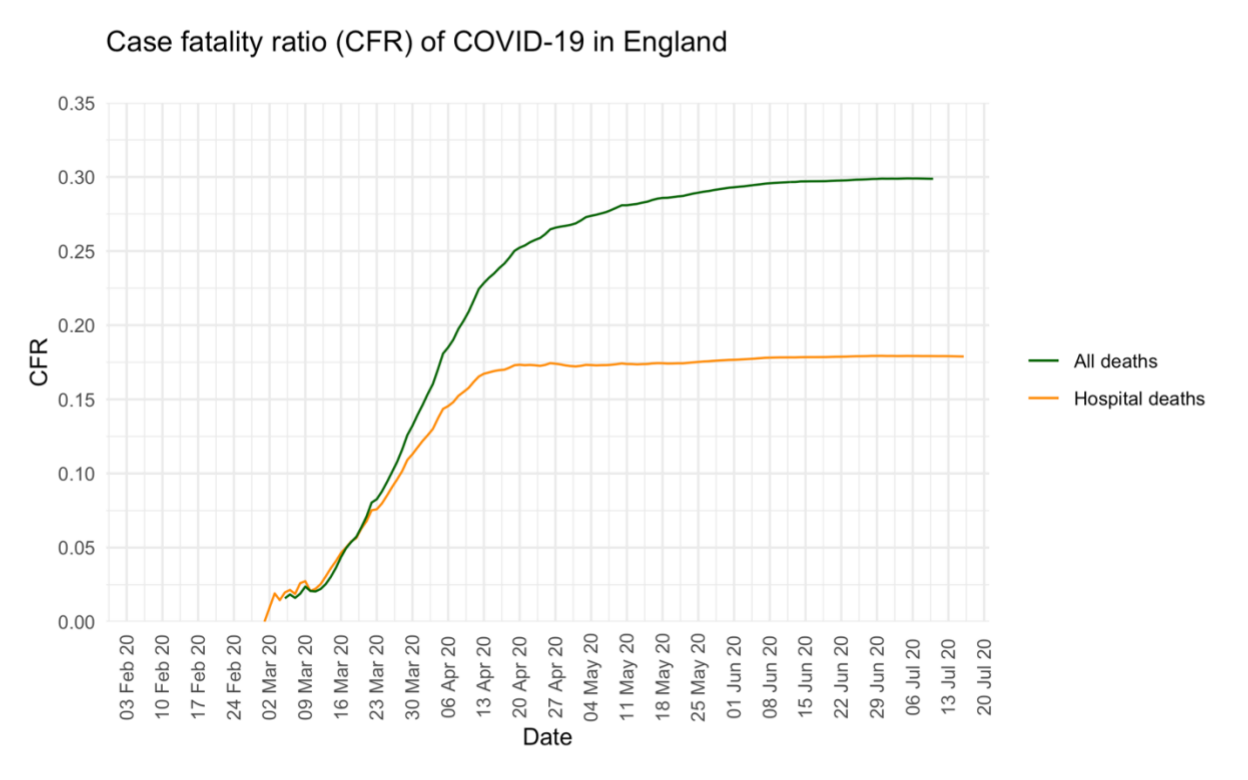

Case fatality ratios (CFRs) in England over time, as calculated using deaths across all settings and hospital deaths only.

Sensitivity analyses

This section describes two sensitivity analyses which were performed.

Inclusion of cases identified by both Pillar 1 and Pillar 2

We explored the sensitivity of our results to the exclusion COVID-19 cases identified by Pillar 2 testing by re-running all analyses using cases identified by both Pillar 1 *and* Pillar 2 of the British government's testing programme.

Table 1 summarises the knot date pairs deemed most likely by this analysis, and Figure 2 displays the observed relationship between cumulative and daily number of new lab-confirmed cases (Pillar 1 and Pillar 2) in England from 30 January to 1 June. The spline models corresponding to the knot date pairs in Table 1 are overlaid on Figure 2.

Figure 3 and Figure 4 show the incident and cumulative lab-confirmed cases of COVID-19 under each stochastically-simulated growth scenario, overlaying the observed data. The results of these simulations are given in Table 2 and Table 3.

These simulations suggest that implementing social distancing and lockdown one or two weeks earlier would have resulted in a 72% or 92% reduction in the total number of cases, respectively, by 1 June. Under the natural history, our model estimated 1897 incident cases on 1 June; this threshold was not exceeded in either counterfactual history. The Poisson deviance

of the natural growth model with respect to incident cases is 5,143, and 5,915 with respect to cumulative cases.

As is evident from these results, the inclusion of Pillar 2 data produces slightly more conservative estimates of percentage reductions in COVID-19 cases numbers, but these remain broadly in line with results from our primary analysis. Additionally, the inclusion of Pillar 2 data creates less well-defined periods of growth, leading to a poorer fitting model overall.

Table 1

Knot date 1 (a)	Knot date 2 (b)	Growth factor 1 $(\alpha_1 + 1)$ [SE]	Growth factor 2 $(\beta_1 + 1)$ [SE]	Growth factor 3 $(\gamma_1 + 1)$ [SE]	Poisson deviance, incident cases	Poisson deviance, cumulative cases	Prob.
24 March	31 March	1.196 [0.044]	1.106 [0.026]	0.988 [0.004]	7760	61303	0.023
24 March	8 April	1.202 [0.046]	1.041 [0.012]	0.984 [0.005]	5294	48270	0.029
24 March	9 April	1.206 [0.048]	1.035 [0.012]	0.984 [0.005]	5044	21289	0.065
24 March	10 April	1.210 [0.050]	1.029 [0.012]	0.985 [0.006]	5393	6815	0.203
24 March	11 April	1.212 [0.051]	1.026 [0.012]	0.985 [0.006]	5828	6974	0.198
24 March	12 April	1.209 [0.052]	1.026 [0.011]	0.984 [0.006]	7760	61303	0.057
24 March	13 April	1.211 [0.051]	1.025 [0.011]	0.984 [0.006]	5294	48270	0.284
24 March	14 April	1.212 [0.050]	1.024 [0.010]	0.983 [0.006]	5044	21289	0.143

Best-fitting pairs of knot dates, with corresponding growth factors (standard errors, SEs) estimated from Eq.2 (main text); all values are given on the normal scale. Poisson deviance with respect to both incident and cumulative cases for the period $1 \leq t \leq T$ are also given. The likelihood-based probability (Prob.) of each pair of knot dates is also given.

Figure 2

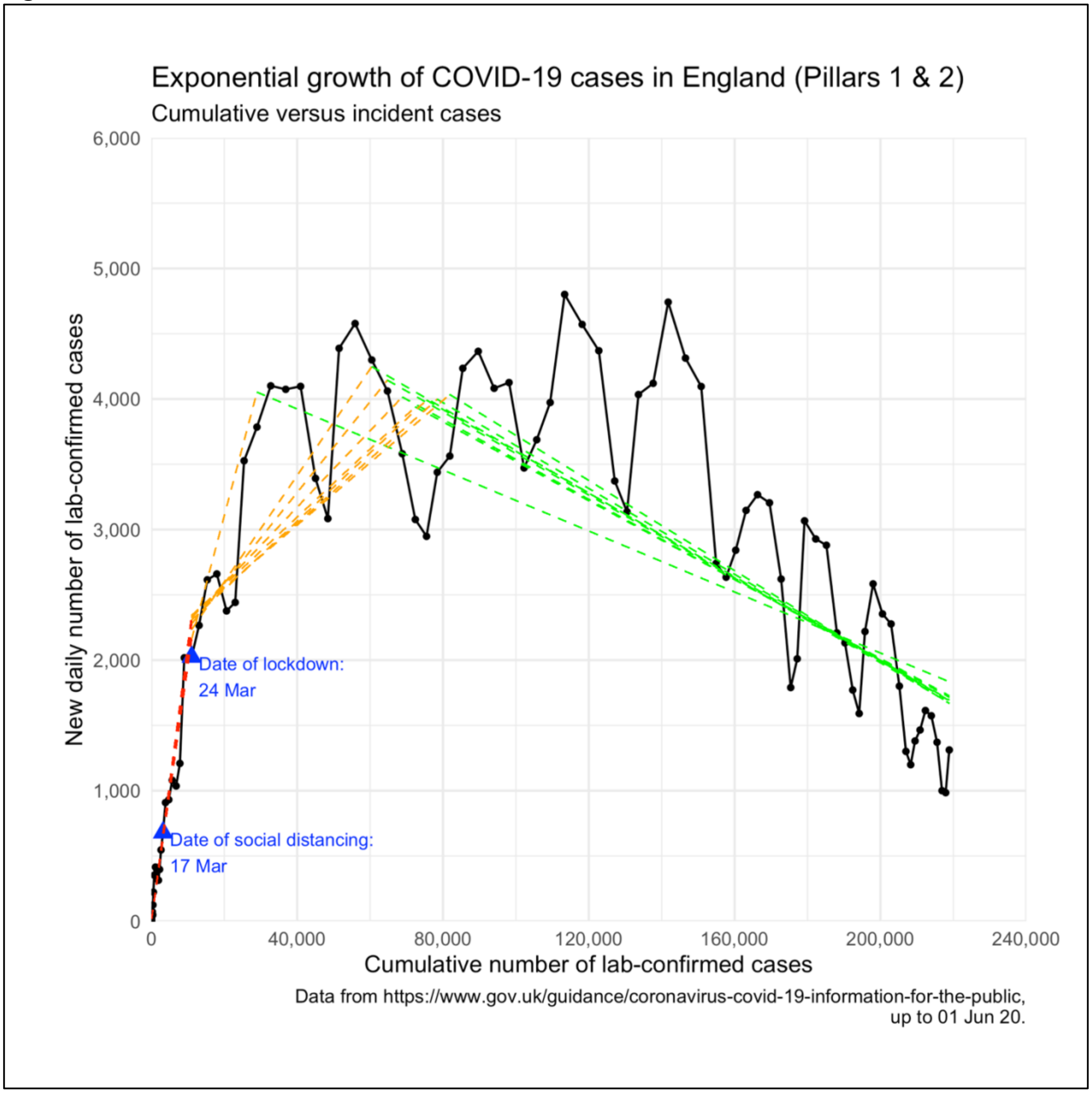

The observed relationship between cumulative and daily number of new lab-confirmed cases (Pillars 1 & 2) in England from 30 January to 1 June. Three distinct growth periods are identified, which may reasonably be attributed to measures implemented by the British government. Growth period 1 (in red) indicates initial uncontrolled growth; growth period 2 (in orange) indicates growth under social distancing; growth period 3 (in green) indicates growth under lockdown. Dates in blue indicate first full days under social distancing and lockdown measures, respectively.

Table 2

Cumulative number of lab-confirmed cases (1 June)			
Natural growth		Counterfactual growth	
Observed	Modelled	Intervention<br>1 week earlier	Intervention<br>2 weeks earlier
220,198	224,137<br>(148,737, 324,721)	62,200<br>(44,486, 84,895)	17,030<br>(13,264, 21,572)

Cumulative number of lab-confirmed cases of COVID-19 in England on 1 June for each scenario modelled. The mean number of cases from 100,000 simulation runs are given for the three modelled scenarios, with 95% simulation intervals (i.e. 2.5 and 97.5 centile estimates) in parentheses.

Table 3

Cumulative number of deaths (1 June)				
	Natural growth		Counterfactual growth	
Deaths<br>(CFR)	Observed	Modelled	Intervention<br>1 week earlier	Intervention<br>2 weeks earlier
Hospital deaths (CFR_1)	27,212	27,699<br>(18,381, 40,129)	7,687<br>(5,498, 10,491)	2,105<br>(1,639, 2,666)
All deaths (CFR_2)	45,130	45,937<br>(30,484, 66,552)	12,748<br>(9,117, 17,399)	3,490<br>(2,718, 4,421)

Cumulative number of deaths resulting from COVID-19 in England on 1 June for each scenario modelled in the sensitivity analysis. Estimates are given according to two separate case fatality ratios: CFR_1, which utilises data from NHS England on deaths occurring in hospitals;[4] and CFR_2, which utilises data from ONS on all deaths.[5] The mean number of deaths from 100,000 simulation runs are given for the three modelled scenarios, with 95% simulation intervals (i.e. 2.5 and 97.5 centile estimates) in parentheses.

Figure 3

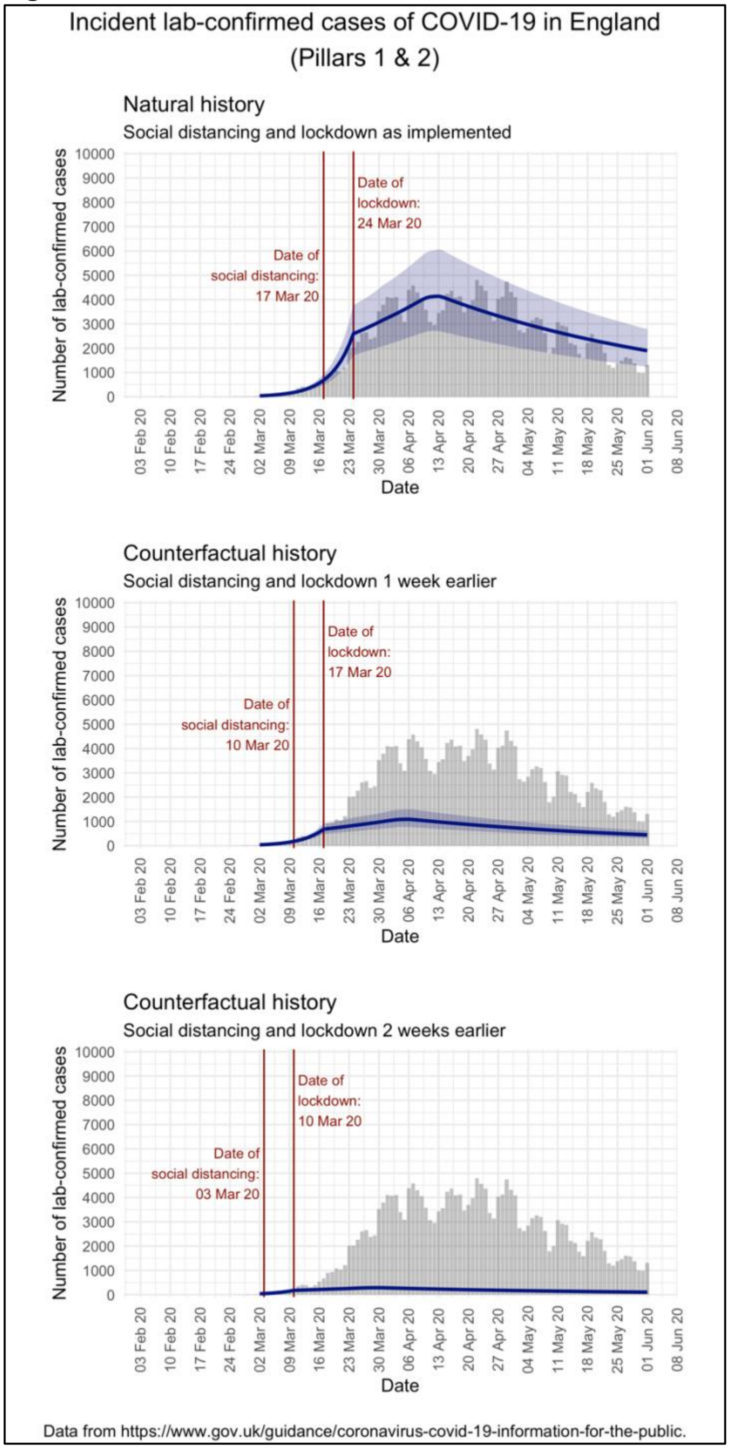

Incident lab-confirmed cases of COVID-19 (Pillars 1 and 2) from 3 March to 1 June, under each of the three scenarios modelled. Indicated social distancing and lockdown dates represent first full days under each measure, respectively. Grey bars represent daily number of observed incident cases. Blue lines represent mean number of incident cases across all 100,000 simulation runs, including the 95% simulation interval.

Figure 4

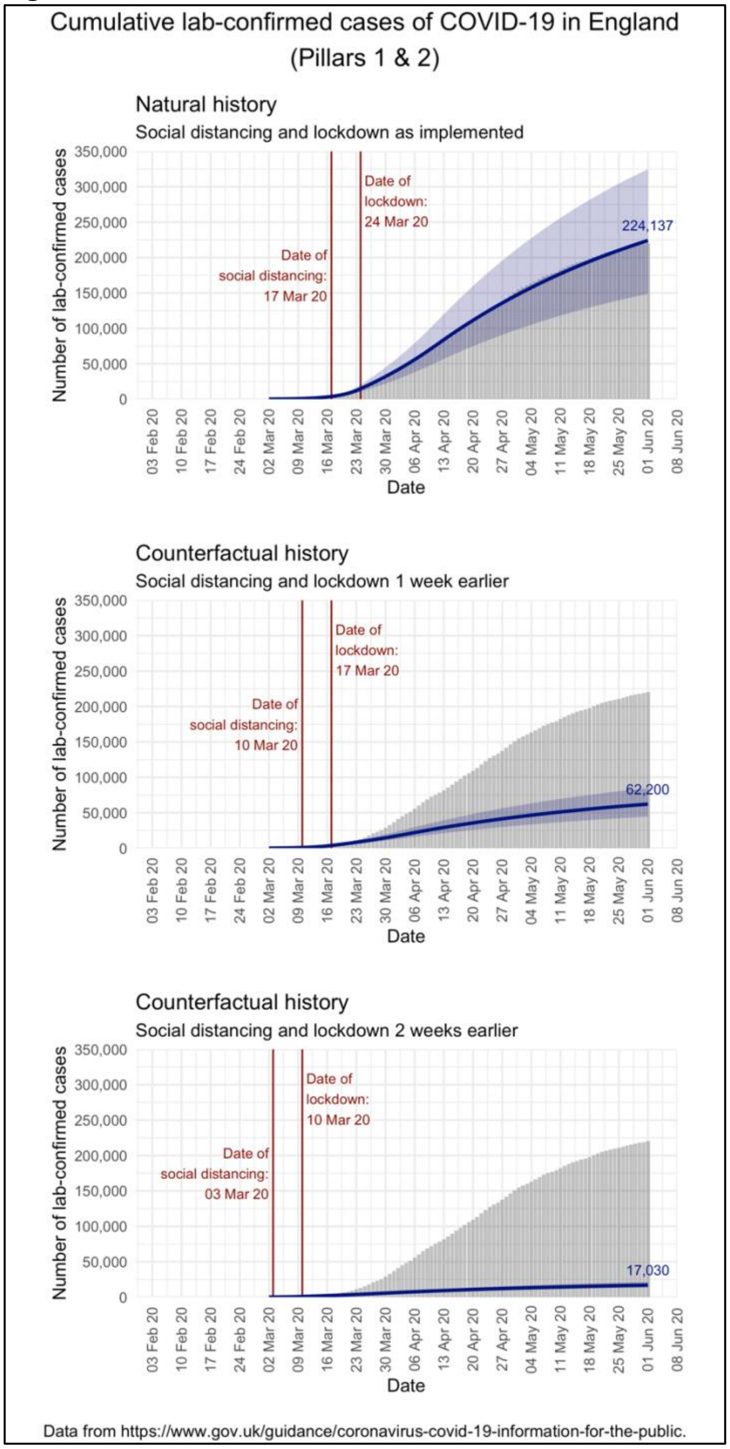

Cumulative lab-confirmed cases of COVID-19 (Pillars 1 and 2) from 3 March to 1 June, under each of the three scenarios modelled. Indicated social distancing and lockdown dates represent first full days under each measure, respectively. Grey bars represent daily number of observed incident cases. Blue lines represent mean number of incident cases across all 100,000 simulation runs, including the 95% simulation interval.

Equal probabilities of best knot date pairs

We explored the sensitivity of our results to the construction of our likelihood-based probability of each knot point pair, by conducting the stochastic simulation in which the probability of each knot point pair was equal (i.e. $prob = 0.2$).

The results of this simulation are given in Table 4 and Table 5. As is evident, the simulation intervals (SIs) for both cases and deaths on 1 June are slightly wider than in the primary analysis, but bottom-line inferences remain unchanged. The Poisson deviance of the natural growth model with respect to incident cases is 2,408, and 2,360 with respect to cumulative cases.

Table 4

Cumulative number of lab-confirmed cases (1 June)			
Natural growth		Counterfactual growth	
Observed	Modelled	Intervention 1 week earlier	Intervention 2 weeks earlier
154,027	157,553 (113,572, 210,425)	40,837 (29,935, 50,484)	10,496 (7,951, 12,735)

Cumulative number of lab-confirmed cases of COVID-19 in England on 1 June for each scenario modelled in the sensitivity analysis. The mean number of cases from 100,000 simulation runs are given for the three modelled scenarios, with 95% simulation intervals (i.e. 2.5 and 97.5 centile estimates) in parentheses.

Table 5

Cumulative number of deaths (1 June)				
	Natural growth		Counterfactual growth	
Deaths (CFR)	Observed	Modelled	Intervention 1 week earlier	Intervention 2 weeks earlier
Hospital deaths (CFR_1)	27,212	27,835 (20,065, 37,176)	7,215 (5,289, 8,919)	1,854 (1,405, 2,250)
All deaths (CFR_2)	45,130	46,163 (33,277, 61,655)	11,965 (8,771, 14,792)	3,075 (2,330, 3,731)

Cumulative number of deaths resulting from COVID-19 in England on 1 June for each scenario modelled in the sensitivity analysis. Estimates are given according to two separate case fatality ratios: CFR_1, which utilises data from NHS England on deaths occurring in hospitals;[4] and CFR_2, which utilises data from ONS on all deaths.[5] The mean number of deaths from 100,000 simulation runs are given for the three modelled scenarios, with 95% simulation intervals (i.e. 2.5 and 97.5 centile estimates) in parentheses.

Code

All simulation and analytical code can be accessed at https://github.com/KFArnold/covid-counterfactual.

A ***README.md*** file is available in the repository, which gives a broad overview of the repository's structure and usage. We also provide detailed notes here regarding how the code functions.

The repository has four main folders:

1. *Data*: This folder contains all cases and deaths data that are used for the analysis.
2. *Code*: This folder contains all code scripts which were used for the analysis.
3. *Results*: This folder contains all outputs/results produced by the analysis.
4. *packrat*: This folder contains all package management files.[8]

The *Code* folder contains four scripts, the main functions of each of which are described below:

1. ***Import and format data.R***
    a. Imports cases and deaths data from the *Data* folder
    b. Formats cases and deaths data for use in all other scripts
    c. Defines important dates for the simulation:
        i. first confirmed case in England
        ii. first full day for which cumulative cases in England exceeded 100 (i.e. $t = 1$)
        iii. end date for simulation (i.e. $t = T$)
        iv. first full day of social distancing
        v. first full day of lockdown
    d. Estimates case fatality ratios (i.e. CFR_1 and CFR_2)
    e. ***Note that this script is called by all other scripts (described subsequently), so that the formatted cases and deaths data, along with important dates and CFR estimates, are accessible to them*
2. ***Descriptive plots.R***
    a. Calls the script *Import and Format Data.R*
    b. Produces descriptive plots and saves them to *Results* folder
        i. Case fatality ratios over time ('*Plot – descriptive – case fatality ratio.png*')
        ii. Incident cases and deaths over time ('*Plot – descriptive – incident cases and deaths.png*')
        iii. Cumulative cases and deaths over time ('*Plot – descriptive – cumulative cases and deaths.png*')
3. ***Identify 2 knot points.R***
    a. Calls the script *Import and Format Data.R*
    b. Defines pairs of potential knot dates
    c. Estimates growth parameters associated with each pair of potential knot dates using an Arima spline model with one autoregressive term (i.e. $p = 1$) and 7-day seasonal adjustment

i. Growth factor 1 (SD) – prior to first knot date (i.e. during initial uncontrolled growth)
ii. Growth factor 2 (SD) – between first and second knot dates (i.e. during social distancing)
iii. Growth factor 3 (SD) – after second knot date (i.e. during lockdown)

d. Simulates growth using each pair of potential knot dates and estimated growth factors, and calculates Poisson deviance between observed (7-day moving average) and predicted incident and cumulative cases
e. Determines best knot point pairs according to Poisson deviance
f. Constructs a likelihood-based probability of each of the best knot point pairs
g. Exports .csv file containing best knot point pairs (including associated growth parameters and probabilities) to *Results* folder ('*Best knot points.csv*')

4. ***Simulation with 2 knots.R***
   a. Calls the script *Import and Format Data.R*
   b. Imports .csv file containing best knot point pairs ('*Best knot points.csv*') from *Results* folder, and defines simulation parameters from this file
   c. Defines scenarios to be simulated (i.e. natural and counterfactual histories)
   d. Simulates growth of cases for both natural and counterfactual histories using best knot points and associated growth parameters
   e. Combines summary results (mean and 95% SI) from all natural and counterfactual histories with observed data and exports .csv files to *Results* folder
      i. Daily cases, for $1 < t \leq T$ ('*Summary – daily cases.csv*')
      ii. Cumulative cases, for $1 < t \leq T$ ('*Summary – cumulative cases.csv*')
      iii. Growth factors, for $1 < t \leq T$ ('*Summary – growth factors.csv*')
      iv. Deaths according to CFR_1 and CFR_2, at $t = T$ ('*Summary – growth factors.csv*')
   f. Creates summary table of cumulative cases and deaths at end of simulation (i.e. $t = T$) and exports .csv files to *Results* folder ('*Final summary – cases and deaths at end of simulation.csv*')
   g. Produces figures and saves the to the *Results* figures
      i. Incident cases under natural and counterfactual histories vs observed data ('*Plot – true vs counterfactual – incident cases.png*')
      ii. Cumulative cases under natural and counterfactual histories vs observed data ('*Plot – true vs counterfactual – cumulative cases.png*')
      iii. Cumulative vs incident cases, with spline models of best knot points overlaid ('*Plot – Cumulative vs incident cases – normal scale.png*')

References

1. UK Department of Health and Social Care. Coronavirus cases in the UK: daily updated statistics. 2020. https://www.gov.uk/guidance/coronavirus-covid-19-information-for-the-public (accessed 22 July 2020.
2. Public Heath England. The weekly surveillance report in England: Coronavirus (COVID-19), week 28 May 2020 to 03 June 2020, 2020.

3. UK Department of Health and Social Care. Coronavirus (COVID-19) cases in the UK. 2020. https://coronavirus.data.gov.uk (accessed 22 July 2020.
4. NHS England. COVID-19 Daily Deaths. 2020. https://www.england.nhs.uk/statistics/statistical-work-areas/covid-19-daily-deaths/ (accessed 22 July 2020.
5. UK Office for National Statistics. Deaths registered weekly in England and Wales, provisional. 21 July 2020 2020. https://www.ons.gov.uk/peoplepopulationandcommunity/birthsdeathsandmarriages/deaths/datasets/weeklyprovisionalfiguresondeathsregisteredinenglandandwales (accessed 22 July 2020.
6. Hyndman R, Athanasopoulos G, Bergmeir C, et al. forecast: Forecasting functions for time series and linear models. R. 8.12 ed; 2020.
7. van der Steen A, van Rosmalen J, Kroep S, et al. Calibrating Parameters for Microsimulation Disease Models:A Review and Comparison of Different Goodness-of-Fit Criteria. *Medical Decision Making* 2016; **36**(5): 652-65.
8. Ushey K, McPherson J, Cheng J, Atkins A, Allaire JJ. packrat: A Dependency Management System for Projects and their R Package Dependencies. R. 0.5.0 ed; 2018.
